# Fréquence hospitalière des crises non épileptiques psychogènes à Bamako

**DOI:** 10.11604/pamj.2024.47.148.42711

**Published:** 2024-03-27

**Authors:** Gaoussou Keita, Joseph Traoré, Souleymane Papa Coulibaly, Kadiatou Traoré, Boubacar Hamadou Maiga, Eloi Aperou Dara, Mahamadou Koné, Zoua Kamaté, Ousmane Soma Diarra, Kassim Diakité, Souleymane Coulibaly, Seybou Hassane Diallo, Youssoufa Mamadou Maiga

**Affiliations:** 1 Département de Psychiatrie, Centre Hospitalier Universitaire Point G, Bamako, Mali,; 2 Faculté de Médecine et d'Odontostomatologie de Bamako, Bamako, Mali,; 3 Université des Sciences, des Techniques et des Technologies de Bamako, Bamako, Mali,; 4 Département de Neurologie, Centre Hospitalier Universitaire Gabriel Touré, Bamako, Mali

**Keywords:** Crises non épileptiques psychogènes, électroencephalogramme intercritique normal, psychiatrie, neurologie, Bamako, Mali, Psychogenic non-epileptic seizures, normal interictal electroencephalogram, psychiatry, neurology, Bamako, Mali

## Abstract

Poser le diagnostic de crise non épileptique est difficile en absence de vidéo-électroencéphalogramme. La commission d´expert de la ligue international contre l´épilepsie propose une démarche diagnostic permettant de poser le diagnostic selon un degré de certitude avec ou en absence de vidéo-électroencéphalogramme. Notre objectif était de déterminer la fréquence hospitalière des crises non épileptiques psychogènes en absence de vidéo-électroencéphalogramme. A l´aide du registre de consultation externe, nous avons identifié les patients suivis pour épilepsie avec deux électroencéphalographies inter-critiques normaux, entre janvier 2020 et octobre 2021. Un examen des dossiers médicaux des patients et une évaluation de la validité du diagnostic ont été effectués. Sur 64 patients évalués avec électroencéphalogramme intercritique normal, 19 ont été inclus comme souffrant de crise non épileptique psychogène, soit 26,68%. La moyenne d´âge était de 23,94 +/- 9,4 ans. Les femmes représentaient 68,4%. Les patients suivis en neurologie ont représenté 84%. Un antécédent de traumatisme dans l´enfance était retrouvé dans (47,4 %). La première crise était précédée d´événements stressants dans 47,36%. Le trouble stress posttraumatique était le plus représenté avec 73,7% des cas. L´âge moyen était de 20,95 +/-9,8 ans pour la première la crise et la durée moyenne d´évolution des crises était de 3 ans +/- 2 ans. Cette étude illustre la possibilité de poser un diagnostic présomptif de crise non-épileptique psychogène en absence de vidéo-EEG.

## Abstract (en)

Diagnosing a non-epileptic seizure is difficult in the absence of a video electroencephalogram. The expert commission of the international league against epilepsy proposes a diagnostic approach allowing the diagnosis to be made according to a degree of certainty with or in the absence of a video electroencephalogram. Our objective was to determine the hospital frequency of psychogenic non-epileptic seizures in the absence of video-electroencephalogram. Using the outpatient registry, we identified patients followed for epilepsy with two normal interictal electroencephalographies, between January 2020 and October 2021. A review of the patients' medical records and an assessment of the validity of the diagnosis were carried out. Out of 64 patients evaluated with normal interictal electroencephalogram, 19 were included as suffering from psychogenic non-epileptic seizures, i.e. 26.68%. The average age was 23.94 +/- 9.4 years. Women represented 68.4%. Patients followed in neurology represented 84%. A history of childhood trauma was found in (47.4%). The first crisis was preceded by stressful events in 47.36%. Post-traumatic stress disorder was the most represented with 73.7% of cases. The average age was 20.95 +/- 9.8 years for the first crisis and the average duration of evolution of the crises was 3 years +/- 2 years. This study illustrates the possibility of making a presumptive diagnosis of psychogenic non-epileptic seizure in the absence of video-electroencephalogram.

## Introduction

Les crises non épileptiques psychogènes (CNEP) sont définies comme des « changements brutaux et paroxystiques de comportement moteurs, des sensations ou de la conscience, qui évoquent de prime abord des crises épileptiques, mais qui ne sont pas liées à une décharge neuronale excessive » [1-3]. Selon le manuel diagnostic et statistique des troubles mentaux dans sa 5^e^ édition (DSM-V), les CNEP sont des crises en rapport avec des processus psychogènes classées dans la catégorie des troubles conversifs [4]. Selon la classification internationale des maladies, 11^e^ révision (CIM 11) les CNEP sont dans la catégorie de trouble dissociatif à symptômes neurologiques, avec convulsions non épileptiques [5]. La suspicion du diagnostic de CNEP est faite devant les arguments cliniques dont la confirmation est donnée par l´EEG/vidéo [6,7]. Le groupe d´expert de la ligue internationale contre l´épilepsie, propose un degré de certitude diagnostic qui dépend du degré avec lequel les CNEP ont été documentées.

Très peu diagnostiquées les CNEP font partie des troubles neurologiques fonctionnels les plus courants [8]. La prévalence du CNEP est difficile à déterminer et n´a pas été directement mesurée. Un long délai entre le début du diagnostic et les patients atteints de CNEP qui se désengagent du suivi médical sont des obstacles considérables aux études épidémiologiques [9]. Selon Lafrance WC Jr *et al*., pour une prévalence de 1% d´épilepsie dans la population des USA, le diagnostic des CNEP est de 5 à 20% [10]. Luciano De Paola *et al*. affirment que les CNEP sont la cause de 20 à 30% des « crises » résistantes à une thérapeutique anti-convulsivante [11]. Les CNEP concernent les femmes pour une proportion de 65 à 80% [3]. Les données en Afrique sur les CNEP sont rares. Cependant on sait par exemple qu´en Afrique du sud, suivant Chrisma Pretotius *et al*., 77% des CNEP étaient des femmes [12]. Au Maroc cette proportion atteignait 93,75% selon Chama R *et al*. [13]. Au Bénin en 2018, Awohouedji M *et al*., dans leur étude, ont trouvé que 84,48% des soignants n´ont pas pu choisir le signe qui permet d´élimer une crise psychogène lors d´une crise d´allure épileptique [14]. A ce jour, aucune étude n'a été menée sur les CNEP au Mali. Le présent travail a eu pour but d´établir la fréquence des CNEP dans les services de psychiatrie du CHU-Point G et de neurologie du CHU-Gabriel Toure.

## Méthodes

Notre étude s´est déroulée dans les services de Psychiatrie du CHU-Point G et de Neurologie du CHU-Gabriel Toure de Bamako.

**Type d´étude:** il s´agissait d´une étude transversale descriptive à recrutement consécutif.

**Période d´étude:** cette étude s´est déroulée sur une période de 22 mois, de janvier 2020 à octobre 2021, dont six (6) mois de collecte de données (mai 2021 à octobre 2021).

**Population d´étude:** notre étude a concerné les patients diagnostiqués épileptiques avec deux EEG normaux et suivis dans les services de psychiatrie du CHU-Point G et de Neurologie du CHU-Gabriel Toure.

**Critères d´inclusion:** nous avons recruté de manière consécutive l´ensemble des patients suivis pour épilepsie avec deux EEG normal ayant accepté l´entretien psychiatrique structuré.

**Critères de non-inclusion:** les patients n´ayant pas accepté de participer à l´étude et ceux qui ne répondaient pas à nos critères.

**Echantillonnage:** nous avons procédé par un échantillonnage consécutif de tous les patients.

**Critère de jugement:** dans cette étude, nous avons défini comme crise non épileptique psychogène selon un rapport de consensus d'experts de la ligue internationale contre l´épilepsie en absence de vidéo-EEG sur des critères de présomption (diagnostic possible) ci-dessus définis: i) l´absence d´activité épileptiforme lors de deux EEG intercritiques de routine; ii) les éléments de l´anamnèse compatible avec une CNEP présence des facteurs de vulnérabilité (prédisposants) des facteurs déclenchants (précipitants), des facteurs de maintien (perpétuants); la description des crises par le clinicien, par le témoin ou par le patient (Tableau 1).

**Tableau 1:** niveaux de certitude diagnostique pour le diagnostic CNEP

Niveau de certitude	Eléments d’anamnése (facteurs biographiques description de ses crises par le patient)	Description de la crise	Examen EEG
Diagnostic possible	Compatibles avec CNEP	Par un témoin ou par auto- description	Absence d’activité épileptiforme lors d’un EEG intercritique de routine ou aprés privation de sommeil
Diagnostic probable	Compatibles avec CNEP	Par un clinicien présent ou ayant lui-même visionné l'enregistrement vidéo de la crise montrant des éléments sémiologiques en faveur de CNEP	Absence d’activité épileptiforme lors d’un EEG intercritique de routine ou aprés privation de sommeil
Diagnostic cliniquement établi	Compatibles avec CNEP	Par un clinicien expérimenté dans le diagnostic des troubles convulsives (vidéo ou présence direct), constatant des éléments sémiologiques en faveur de CNEP sans EEG concomitant	Absence d’activité épileptiforme lors d’un EEG intercritique ou d’un EEG au cours d’une crise atypique dont la sémiologie pourrait laisser présager une activité électrique épileptiforme
Diagnostic documenté	Compatibles avec CNEP	Par un clinicien expérimenté dans le diagnostic des troubles convulsives (vidéo ou présence direct), constatant des éléments sémiologiques en faveur de CNEP avec EEG concomitant	Absence d'activité épileptiforme avant, au cours ou immédiatement aprés une crise enregistrée en vidéo-EEG avec présence d’éléments sémiologiques en faveur de CNEP

**Collecte des données:** les données ont été recueillies sur une fiche d´enquête préétablie. La collecte consistait à repérer les cas potentiel à partir des dossiers cliniques, le registre d´enregistrement des patients, la description des crises par le patient, le témoin, les cliniciens des services concernés par l´étude. Ensuite, une rencontre duelle était organisée avec les candidats potentiels à l´étude. Lorsqu´il était établi que le candidat répond aux critères d´inclusions, il était soumis selon son approbation au guide d´entretien. Dans un deuxième temps le candidat était soumis à une évaluation psychopathologique à l´aide des outils (L´échelle de dépression et d´anxiété de Hamilton, L´échelle PCL-5, Le SDQ).

**Variables étudiées:** nous avons étudié : i) les caractéristiques sociodémographiques: age, sexe, niveau d´études, profession, niveau socioéconomique, profession, statut marital, résidence, lieu de vie; ii) les antécédents personnels et familiaux; iii) l´histoire de la maladie; iv) les caractéristiques cliniques.

**Saisie et analyse des données:** les données ont été saisies et analysées à l´aide du pack office world, du logiciel SPSS; les références bibliographiques à partir du logiciel Zotero.

## Résultats

**Taille de l´échantillon:** nous avons dépouillé un total de 77 dossiers-patients avec EEG intercritiques négatifs, 64 patients ont été évalués et nous avons inclus 19 patients comme soufrant de CNEP. Ainsi la prévalence des CNEP parmi les patients suivis pour épilepsie avec EEG normal était de 19/64 soit 29,68%.

**Caractéristiques sociodémographique:** la moyenne d´âge des patients était de 23,94 +/- 9,4 ans avec des extrêmes allant de 10 à 50 ans. Le sexe féminin était le plus représenté avec 12/19 cas (68,4%). Les patients célibataires représentaient 11/19 cas (soit 57,9%). Les patients nés de parents mariés représentaient 13/19 cas (soit 68,4%). Les patients de la population sans aucune activité professionnelle représentaient 12/19 cas (soit 63%). Les patients vivaient pour la plupart en milieu urbain avec 11/19 cas (57,9%), avec leur(s) conjoint(es) - 7/19 cas (36,8%), sous la coupe des deux parents dans 5/19 cas (soit 26,3%), confié à un membre de la famille dans 4/19 cas (21,1%). Les patients de niveau scolaire primaire étaient les plus représentés avec 7/19 cas (36,8%) (Tableau 2).

**Tableau 2:** caractéristiques sociodémographiques de patients avec diagnostic probable de crises non épileptiques psychogènes

Variable	N	%
**Age**	**19**	
10 ans à 16 ans	6	31,6
18 ans à 30 ans	9	47,4
31 ans à 50 ans	4	21,1
**Sexe**	**19**	
Masculin	6	31,6
Féminin	13	68,4
**Statut maritale**	**19**	
Célibataire	11	57,9
Marié	7	36,8
Séparé/divorcé	1	5,3
**Situation matrimoniale des parents**	**19**	
Marié	13	68,4
Divorcé /séparé	3	15,8
Jamais marié	3	15,8
**Activité professionnelle**	**19**	
Régulière	5	26,3
Irrégulière	2	10,5
Absente	12	63,2
**Milieu de vie**	**19**	
Urbain	11	57,9
Rural	6	31,6
Semi urbain	2	10,5
**Vie communautaire**	**19**	
Seul	1	5,3
Avec les deux parents	5	26,3
Avec la mère seul	1	5,3
Avec le pére seul	1	5,3
Avec conjoint (es)	7	36,8
Confié	4	21,1
**Niveau scolaire**	**19**	
Jamais scolarisé	2	10,5
Primaire	7	36,8
Secondaire	5	26,3
Universitaire	5	26,3

**Lieu de suivi et contexte d´hospitalisation:** la majorité des patients était suivie en neurologie avec 16/19 cas (soit 84,2%). L´hospitalisation a concerné 3 patients de la population étudiée (15,8%) entre 2017 et 2020, en moyenne 2,6 fois avec une durée moyenne de séjour hospitalier de 114, 67 jours. Les motifs de l´hospitalisation étaient dans 2/19 cas (soit 10,5%) une impotence fonctionnelle des membres inférieurs, 1/19 (soit 5,3%) une crise épileptiforme. Nos patients avaient fait leurs premières consultations en neurologie dans 16/19 cas (84,2%) (Tableau 3).

**Tableau 3:** lieu de suivi des patients et contexte d´hospitalisation

Variable	N	%
**Service**	19	
Psychiatrie	3	15,8
Neurologie	16	84,2
**Mode de suivi**	**19**	
hospitalisation	3	15,8
Consultation ambulatoire	16	84,2
**Date de la Première hospitalisation**		
13.11.2017	1	5,3
21.02.2020	1	5,3
18.03.2020	1	5,3
**Lieu de la première hospitalisation**		
neurologie	1	5,3
psychiatrie	2	10,5
**Motif de la première hospitalisation**		
crise èpileptiforme	1	5,3
impotence fonctionnel	2	10,5
**Nombre de fois d'hospitalisation**		
1	1	5,3
3	1	5,3
4	1	5,3
**Durèe cumulèe de jours d’hospitalisation**		
14	1	5,3
90	1	5,3
240	1	5,3
**Lieu de la 1ère consultation et Profil du soignant**	19	
Psychiatrie	2	10,5
Neurologie	**16**	**84,2**
Gènèraliste	1	5,3

**Antécédents personnels généraux:** un traumatisme crânien avait été retrouvé chez 3/19 patients (15,8%). La tentative de suicide avait été retrouvée dans 1/19 cas (5,3%). La consommation de substances psychoactives avait été retrouvée chez 3/19 des patients de la population étudiée (15,9%). Le trouble alimentaire à type d´anorexie était identifié dans 6/19 cas (31,6%). Les patients scolarisés de la population étudiée ont eu des difficultés scolaires dans 7/18 cas (36,8%) (Tableau 4).

**Tableau 4:** antécédents personnels généraux des patient CNEP

Variable	N	%
Antécédents traumatisme crânien	**19**	
Oui	3	15,8
Non	16	84,2
**ATCD de tentative de suicide**	**19**	
Oui	1	5,3
Non	18	94,7
**Consommation de substance psychoactif**	**19**	
Tabac	1	5,3
Alcool	1	5,3
Anxiolytique	1	5,3
Néant	16	84,2
**Trouble alimentaire**	**19**	
Anorexie	6	31,6
Aucun	13	68,4
**Difficulté scolaire**	**19**	
Oui	7	36,8
Non	11	57,9
Jamais scolarisé	1	5,3

**Antécédents personnels d´événements de vie stressants:** le traumatisme dans l´enfance avait été retrouvé dans 9/19 cas (47,4 %), dont 6 cas de maltraitances psychiques, 2 cas de négligences parentales et 1 cas d´agression sexuelle. La violence familiale était l´événement traumatique la plus représenté avec 42,8% (6/14). Les crises avaient succédé à un événement stressant dans 9/19 cas (47,4%), dont 4 cas de deuils, 3 cas de séparations et 2 cas d´accidents graves de la circulation (Tableau 5).

**Tableau 5:** antécédents personnels d´événements de vie stressants

Variable	N	%
Traumatisme dans l´enfance	**19**	
Présent	9	47,4
Absent	10	52,6
**Type de traumatisme dans l’enfance**	**9**	
Agression sexuelle	1	5,3
Maltraitance psychique	6	31,6
Négligence parentale	2	10,5
Maltraitance physique	0	0
**Evénements traumatiques sur toute la vie**	**19**	
Violence familiale	6	42,8
Deuil	3	14,2
Viol	1	7,1
Situation de guerre	2	14,2
Séparation précoce avec les parents	2	14,2
Non connu	5	26,3
**Evénement stressant précédent l’apparition de la premiére crise**	**19**	
Deuil	4	21,1
Séparation	3	15,8
Accident grave (AVP)	2	10,5
Néant	10	52,6
**Antécédent familiaux de crise épileptique et épileptiforme**		
Variable	N	%
Epilepsie	4	21,1
Crise épileptiforme dite «djinèbana»	5	26,3
Néant	10	52,6
**Total**	19	100

**Evaluation à l´échelle psychométrique:** selon l´évaluation psychométrique 17/19 patients (soit 89,5%) avaient un score d´anxiété et de dépression de Hamilton supérieur à 10; 14/19 patients (73,7%) avaient un score d´échelle PCL-5 supérieure à 33; 14/19 (73,7%) avaient un score de SDQ20 supérieur à 31 (Tableau 6).

**Tableau 6:** évaluation à l´échelle psychométrique

Variable	N	%
Echelle d´anxiété et dépression de Hamilton	**19**	
Etat anxieux ou dépressif absent	2	10,5
Etat anxieux ou dépressif certain	17	89,5
Echelle PCL-5	**19**	
TSPT -	5	26,3
TSPT +	14	73,7
Echelle SDQ20	**19**	
Tendance dissociative -	5	26,3
Tendance dissociative +	14	73,7

**Antécédents familiaux de crise épileptique et épileptiforme:** les patients avaient un antécédent familial de crises épileptiformes dites « djinébana » dans 5/19 cas (26,3%) et 4/19 cas (21,1%) d´épilepsie confirmée (Tableau 5).

**Histoire et caractéristiques cliniques de la maladie:** l´âge moyen de début des crises des patients étaient de 20,95 +/- 9,8 ans avec des extrêmes 10 ans et 49 ans. La durée d´évolution des crises était estimée entre 2 et 5 ans dans 10/19 cas (52,6%) avec une moyenne de 3,21 ans +/- 1 an. Le mode de début était: brutal chez 12/19 (63,2%), progressive chez 7/19 des patients (36,8%). Les crises généralisées étaient les plus représentées avec 16/19 cas (84,21%). Un facteur déclenchant avait été retrouvé dans 17/19 cas (89,5%), dont 8/19 cas de stress, 6/19 cas de frustration et 3/19 cas de fatigue (Tableau 7).

**Tableau 7:** histoire et caractéristiques cliniques de la maladie

Variable	N	%
Durée d´évolution des crises	**19**	
0 à 3 mois	1	5,3
4 à 1 an	1	5,3
2 ans à 5 ans	10	52,6
Plus de 5 ans	7	36,8
Mode de début des crises	19	
Brutal	12	63,2
Progressive	7	36,8
Type de crise	19	
Généralisé tonico-clonique	13	68,4
Tremblement excessif des membres	1	5,3
Crise catatonique rigide	2	10,5
Absence avec rupture de contact	3	15,8
Facteurs déclenchants	19	
Stress	8	42,1
Frustration	6	31,6
Fatigue	3	15,8
Absent	2	10,5

## Discussion

Nos résultats montrent en outre qu´il est possible de poser le diagnostic présomptif de CNEP en absence de vidéo-EEG en adoptant les recommandations du rapport de consensus d'experts de la ligue internationale contre l´épilepsie. Il existe de nombreux « signaux d´alarme » [15]. La CNEP semble être un problème commun aux épileptologues, mais il n´y a pas de bonne estimation de leur prévalence dans la population générale [16]. Notre étude avait rapporté une fréquence hospitalière de 26,68 %. Lafrance WC Jr *et al*. avaient estimé que pour une prévalence de 1% d´épilepsie dans la population des USA, le diagnostic des CNEP était évoqué pour 5 à 20% [10]. Ailleurs une étude norvégienne sur la population générale rapporte une prévalence de 4,1/100000 habitants [17]. Renato L *et al*. [18] dans leur étude sur l´évaluation des patients suspectés de CNEP en vidéo-EEG intensive avaient trouvé une fréquence de 50% avec 34,6% d´épilepsie associé. Nous estimons que notre fréquence est sous-estimée étant donné l´exclusion des patients présentant une épilepsie confirmée par les critères cliniques. Dans notre étude, la majorité des patients était jeune avec une moyenne d´âge 23,94 +/- 9,4 ans. Cette valeur est proche des études de Coraline H *et al*. 23,94 +/- 9,4 ans [19], de Asadi A *et al*. 23,2 ans +/- 10,1 [20] et de Patidar Y *et al*. 25,44 +/- 10,22 ans [21]. Nous pouvons donc en conclure que les CNEP ont un début d´âge assez tardif compris entre la deuxième et troisième décennie de vie. La majorité de l'échantillon CNEP de notre étude était composée de femmes 68,4%. Cette tendance est conforme à celle d'autres études, notamment Asadi A *et al*. [20] (66,8%), Chrisma Pretorius *et al*. [12] (77%), Luciano De Paolo *et al*. [11] (78%). Elle est nettement plus élevée dans les études de Maria Marta Areco Pico *et al*. [22] (90,46%), de Chama R *et al*. [13] (93,3%). Les études de Bora *et al*. [23], A. Deveci *et al*. [24] ont rapporté que la CNEP est liée au sexe et plus fréquemment observée chez les femmes. La nature exacte de la relation étroite entre les femmes et la CNEP n'est pas encore bien comprise. Bompaire F *et al*. [25], T Van Merode *et al*. [26] dans leurs études n´ont pas trouvé de différence entre les sexes quant à l´apparition des CNEP. Nous ne pouvons pas affirmer la différence de proportion des CNEP entre les femmes et les hommes comptent tenue de la taille de notre échantillon.

Selon le statut marital, les patients dans notre étude semblent être un groupe hétérogène, 58% étaient célibaires, 37% étaient mariés, alors que 5% étaient divorcés. Ces données sont proches de celles de l´étude de Carton S *et al*. [27] (célibataire 56%, Marié(e) 39%, Divorcé/séparé 5%). Des données différentes ont été rapportées dans les études de A. Asadi *et al*. [20] (45% célibataires, 51,2% mariés, 2,4% divorcés), Coraline H *et al*. [28] (42.1% n´avaient jamais vécu en couple, 31.6 % vivaient en couple ou sont mariés, 26.3% étaient séparés ou divorcés). Les CNEP sont confondues avec l´épilepsie qui est culturellement considérée comme une pathologie honteuse. Ainsi nous pensons que le poids de la CNEP diminue les chances des patients de se mettre et ou de se maintenir en couple. La majorité de nos patients 68% sont issues de parents mariés et 32% de parents divorcés, jamais mariés. Un taux inférieur est rapporté en Chine dans l´étude de An DM *et al*. [29] 4,7% des patients sont nés de parents mariés. Cette différence peut être liée à la représentation socio-culturelle du mariage dans notre société, son caractère collectif et sociétal et le devoir des parents de légitimer leurs progénitures. Plus de la moitié des patients de notre étude n´avaient aucune activité professionnelle 63% ; 10,5% avaient des activités irrégulières. Ces données sont proches de celles de l´étude de Chama R *et al*. [13] avec 62,6% sans emplois et 6% qui avaient une activité irrégulière. Il en est de même de l´étude de Coraline H *et al*. [28] où près de 58% des patients étaient sans emploi. La récurrence des crises CNEP fait perdre les chances d´apprentissage et d´employabilité des patients en rapport avec son rapprochement à l´épilepsie qui est une pathologie stigmatisée et discriminant.

La majorité de nos patients 57,9% vivaient dans le milieu urbain, 32,1% en milieu rural et semi-urbain. Chama R *et al*. [13] dans leurs études ont trouvé une proportion plus élevée en milieu urbain 81,3% et 18,8% en milieu rural. Cet état de fait pourrait s´expliquer par la concentration des habitants et les structures spécialisées dans le milieu urbain. Par ailleurs, pour des raisons d´accessibilité, certains patients ont été confiés à des proches vivants en milieu urbain. La majorité des patients de notre étude étaient scolarisée avec une proportion élevée du niveau primaire à secondaire et une faible proportion des universitaires, ce constat est fait dans la majorité des études [20,28]. La difficulté scolaire avait été retrouvée chez 36,8%. Nous pensons que les perturbations de cursus scolaire pourraient s´expliquer par le pic de pourcentage des CNEP chez les moins de 30 ans coïncidant ainsi aux années d´études. Bien que les CNEP soient psychogènes des antécédents somatiques ont été rapportés par beaucoup d´études [29,30]. Dans notre étude, 15,8% des patients CNEP présentaient un antécédent de traumatisme crânien. Ce résultat est proche de l´étude de An DM *et al*. [29] 20,3%; pourcentage plus élevé dans l´étude de LE Westbrook *et al*. [30] 32%. Cette différence pourrait s´expliquer par la faite que LE Westbrook a étudié les CNEP chez les patients traumatisés crâniens. La tentative de suicide avait été rapportée chez un patient. Dans la littérature, on retrouve des taux de tentative de suicide plus élevés, telle que l´étude de Bora *et al*. [23] 6%, celle de A. Bout *et al*. [31] 33%. Nous pensons que la tentative de suicide demeure un tabou dans notre société et est peu exprimée. L´addiction aux substances psychoactives avait été retrouvée chez 3 patients (15,7%) ; respectivement au tabac, à l´alcool ou à l´anxiolytique, ce dernier étant consécutif à une prescription médicale pour anxiété. Chama R *et al*. [13] ont trouvé 6% de dépendance au tabac et au cannabis et Patidar Y *et al*. [21] 4,76% de dépendance à l´alcool et de toxicomanie. Les substances psychoactives pourraient être consommés à bute anxiolytique dans les CNEP. Dans notre étude 47,4% des patients avaient des antécédents psychiatriques familiaux dont 26,3% de crise épileptiforme «djinèbana» (psychogène). Une épilepsie familiale confirmée a été retrouvé chez 21,1% de nos patients. D´autres auteurs ont retrouvé des notions d´épilepsie familiale, ou dans l´environnement familiale, comme: Kette Dualibi R *et al*. [32] 37,6%; Asadi A *et al*. [20] 33,1% et Coraline H *et al*. [28] 31,57%.

La notion d´épilepsie ou de crise épileptiforme dans la famille était importante à rechercher. Les patients peuvent être témoins de crises épileptiques et reproduire inconsciemment ces symptômes [33]. Nous pensons que les patients CNEP pourraient investir ces crises comme mode de décharge et/ou d´expression de leurs angoisses. La majorité des patients CNEP de notre échantillon avait fait leurs premières consultations en neurologie pour crise épileptique dans 84% des cas. Contrairement à l´étude de Chama R *et al*. [13], qui avait rapporté que 81,3% des patients étaient suivi par un psychiatre et ont été référés à un neurologue pour le diagnostic différentiel de leurs crises. Nous estimons que cet état de fait est probablement lié à l´inexistence de service tertiaire en épileptologie multidisciplinaire dans notre pays d´une part, d´autre part par la stigmatisation du service de psychiatrie liée aux représentations socioculturelles. La littérature rapporte que les patients CNEP sont les plus gros consommateurs du système sanitaire d´urgence et sont hospitalisés le plus souvent avec un séjour cumulé d´hospitalisation assez élevé [2,27]. Les patients de notre échantillon ont peu utilisé les services d´urgences en lien avec l´itinéraire thérapeutique qui commence avec les tradithérapeutes. La durée moyenne de séjour d´hospitalisation avait été de 114, 67 jours. Selon Freud. S « *Le séjour long pourrait s´expliqué par la difficulté de poser le diagnostic où l´atténuation d´un symptôme faisait apparaître un autre par un processus psychologique complexe*» [33]. L´anorexie a été le trouble alimentaire retrouvé chez 31,6% de nos patients. Il s´est agi d´un dégout alimentaire précédant ou succédant à la crise durant plusieurs jours. Ce résultat est superposable à celui de Chama R *et al*. [13] (31,25%). Dans notre étude 47,4% des patients avaient subi un traumatisme dans l´enfance. Nos résultats sont, à ce sujet, proches des études de Bora *et al*. [23] et de Chama R *et al*. [13] respectivement 43% et 40,6% de traumatisme dans l´enfance. La corrélation traumatisme dans l´enfance et CNEP a été rapportée dans beaucoup d´études [24,29].

La maltraitance psychique a été le traumatisme dans l´enfance le plus fréquent dans notre étude avec 31,6% des cas. Ce résultat est proche des études de Dong-mei An *et al*. [2,8] et de Patidar Y *et al*. qui avaient rapporté respectivement un taux de 25% de traumatismes psychologiques. Par contre, l´étude de Chama R *et al*. [13] avait rapporté un taux plus bas de 18,75% de maltraitance psychique. La maltraitance physique n´a pas été signalée par les patients de notre étude, cependant la littérature rapporte des taux de maltraitance physique comme dans les études de Coraline H *et al*. [20], de Chama R *et al*. [13] et de Asadi A *et al*. [19] respectivement 10,5%, 25% et 32,4%. Cette différence pourrait s´expliquer par la définition opérationnelle de la maltraitance physique dans notre étude (sévices corporels ayant entrainé des blessures physiques). En ce qui concerne l´agression sexuelle, dans notre étude nous avons trouvé un cas d´agression sexuelle dans l´enfance. Dong-mei An *et al*. [28] en Chine, dans leur étude, ont rapporté 0 cas; celui de Asadi A *et al*. en Iran [19] 8% et de Chama R *et al*. au Maroc [13] 18,8%. Ailleurs, des taux plus élevés sont rapportés, notamment dans l´étude de Coraline H *et al*. en France [20] 42,1% et de celui de Balet MG *et al*. au USA [34] 58,5%. Ces variations de taux d´agressions sexuelles pourraient s´inscrire dans les différences socioculturelles entres les pays, en effet tous ce qui s´apparente au sexe reste un tabou. Il a été rapporté dans la littérature que la survenue des CNEP succède à des événements stressant dans les mois ou années précédents [6,14,23]. Dans notre étude, 47,4% des patients CNEP avaient eu un événement stressant précédent la survenue des crises, ce qui est proche des études de A Deveci *et al*. [23], et de Chama R *et al*. [13] qui ont rapporté respectivement 49,6% et 53,1% d´événements stressants. Nous postulons à l´instar de beaucoup d´autres auteurs dans la littérature que la gestion du stress pourrait jouer un grand rôle dans le déclanchement et la pérennisation des CNEP [3,7,8,26]. Dans la littérature, plus de la moitié des patients CNEP présente une comorbidité psychiatrique allant de 68,8% à 73,5% [13,30]. Tous les patients CNEP de notre étude présentaient une comorbidité psychiatrique. Le diagnostic le plus fréquent dans notre étude était l´état de stress post traumatique (73,7%), ce résultat est supérieur aux études de A. Bout C *et al*. [32] et de K. Mondon *et al*. [35] qui avaient rapporté respectivement 18,6%, 35,13%).

D´autres études comme celles de Ronak Dixit *et al*. [30] et An DM *et al*. [29], ayant un intérêt pour l´anxiété-dépression, rapportent une comorbidité anxiété, dépression, respectivement de 65,8% et 90,16%. Cet écart de résultats pourrait s´expliquer par les outils d´évaluations utilisés et l´intérêt de la recherche. L´âge moyenne de début des crises dans notre étude était de 20,95 +/- 9,8 ans. Nos résultats rejoignent les études de An DM *et al*. [29] 20,7 +/- 14,9 ans; de Chama R *et al*. [13] 23,97 +/- 9,75 ans et de Roderick Duncan *et al*. [36] 30,5±13,7 ans. Ces résultats confirment le début d´apparition assez tardive des CNEP décrit dans la littérature [23,37,38]. La moyenne d´évolution des crises était de 3,21 ans +/- 1 an. Ce résultat est proche de l´étude de Chama R *et al*. 2016 [13] qui avait rapporté une moyenne d´évolution des CNEP de 5,25 +/- 4,91 ans. Cependant, dans l´étude de Roderick Duncan *et al*. [36] composée de beaucoup plus d´adultes, la moyenne d´âge d´évolution des CNEP était plus élevée 37,2±13,7 ans. La moyenne d´évolution relativement courte de notre étude pourrait s´expliquer par la composition de notre échantillon faite d´adolescents et d´adultes. Il conviendrait de conclure que le délai d´apparition de la CNEP et son diagnostic est beaucoup plus long [21,39]. Les CNEP se caractérisent par un début progressif [30]. Cependant notre étude a rapporté un mode de début brutal chez 63,2% des patients, et progressif chez 36,8% des patients. Nos données sont superposables à l´étude de Chama R *et al*. [13] rapportant un mode de début des crises brutal chez 68,8% de ces patients, et progressif chez 31,3%.

Ce constat pourrait s´inscrire dans la difficulté des patients et de leurs familles de faire le lien entre l´apparition de certains symptômes et l´installation de la crise. Les crises généralisées étaient décrites par plus de la moitié des patients CNEP de notre échantillon 84,21%. Ce résultat est proche de l´étude de Chama R *et al*. [13] et de Oto M *et al*. [40] qui avaient décrit chacun 71,9% de crises généralisées. L´aspect spectaculaire de la crise généralisée marqué par la chute et la perte de connaissance et son cortège d´inquiétudes faisant craindre une cause organique, conduisent à une consultation médicale, expliquant le pourcentage élevé des crises généralisées décrit par les patients CNEP de notre échantillon. Dans la démarche diagnostique psychiatrique, les facteurs de stress occupent une grande place dans une vision biopsychosociale, soit comme facteur prédisposant, précipitant ou perpétuant [41,42]. La majorité des patients CNEP de notre étude avait rapporté l´existence de facteurs déclenchants 89,5%. Ce résultat est superposable à ceux de Selkirk *et al*. [4,2] et d´Oto M *et al*. [40] avec respectivement 70,3% et 61,9% des patients qui décrivent l´existence de facteur déclenchant. Nous pensons que ces facteurs déclenchants joueraient un rôle précipitant mais aussi perpétuant par un mécanisme de rappel traumatique en lien avec la mémoire traumatique et la mobilisation émotionnelle. Cela, d´autant plus que le stress était le facteur déclenchant le plus rapporté, soit 42,1% de nos patients. Chama R *et al*. [13] avaient rapporté 32,3% de stress.

## Conclusion

Cette étude nous avait montré une prévalence hospitalière de 29,68% parmi les patients suivis pour épilepsie avec EEG normal. La moyenne d´âge des patients était de 23,94 +/- 9,4 ans. Les femmes étaient les plus représentées avec 68,4%. Les données de cette étude basée sur des critères cliniques en absence de vidéo-EEG et la taille de notre échantillon doivent être considérés avec prudence et nécessitent d´être validés sur une population plus large.
